# Genotyping of the Major SARS-CoV-2 Clade by Short-Amplicon High-Resolution Melting (SA-HRM) Analysis

**DOI:** 10.3390/genes12040531

**Published:** 2021-04-05

**Authors:** Hector Diaz-Garcia, Ana L. Guzmán-Ortiz, Tania Angeles-Floriano, Israel Parra-Ortega, Briceida López-Martínez, Mirna Martínez-Saucedo, Guillermo Aquino-Jarquin, Rocío Sánchez-Urbina, Hector Quezada, Javier T. Granados-Riveron

**Affiliations:** 1 Molecular Pathogenesis Research Laboratory, Hospital Infantil de México Federico Gómez, Mexico City 06720, Mexico; hecdiazgar@gmail.com (H.D.-G.); ketoka10@hotmail.com (M.M.-S.); roci0404@gmail.com (R.S.-U.); 2 Laboratory of Research in Immunology and Proteomics, Hospital Infantil de México Federico Gómez, Mexico City 06720, Mexico; lauris_26san@hotmail.com (A.L.G.-O.); tania.angeles@yahoo.com.mx (T.A.-F.); hquezada@himfg.edu.mx (H.Q.); 3 Clinical Laboratory, Hospital Infantil de México Federico Gómez, Mexico City 06720, Mexico; i_parra29@hotmail.com (I.P.-O.); brisalopezmtz@gmail.com (B.L.-M.); 4 Laboratory of Research in Genomics, Genetics and Bioinformatics, Hospital Infantil de México Federico Gómez, Mexico City 06720, Mexico; guillaqui@himfg.edu.mx

**Keywords:** SA-HRM, allele-specific RT-PCR, SARS-CoV-2 clades, genotyping

## Abstract

The genome of the SARS-CoV-2 virus, the causal agent of the COVID-19 pandemic, has diverged due to multiple mutations since its emergence as a human pathogen in December 2019. Some mutations have defined several SARS-CoV-2 clades that seem to behave differently in terms of regional distribution and other biological features. Next-generation sequencing (NGS) approaches are used to classify the sequence variants in viruses from individual human patients. However, the cost and relative scarcity of NGS equipment and expertise in developing countries prevent studies aimed to associate specific clades and variants to clinical features and outcomes in such territories. As of March 2021, the GR clade and its derivatives, including the B.1.1.7 and B.1.1.28 variants, predominate worldwide. We implemented the post-PCR small-amplicon high-resolution melting analysis to genotype SARS-CoV-2 viruses isolated from the saliva of individual patients. This procedure was able to clearly distinguish two groups of samples of SARS-CoV-2-positive samples predicted, according to their melting profiles, to contain GR and non-GR viruses. This grouping of the samples was validated by means of amplification-refractory mutation system (ARMS) assay as well as Sanger sequencing.

## 1. Introduction

SARS-CoV-2 caused the ongoing pandemic severe respiratory coronavirus disease 2019 (COVID-19), which was reported for the first time in China in December 2019 [1]. During its replication, SARS-CoV-2 can undergo mutation, a change in the sequence of its genome. In turn, genomes that differ by one or more mutations are called variants. A clade is a group of variants that share a common ancestor, whereas a strain is a variant with a distinctively different phenotype [2,3]. A dynamic lineage nomenclature has been adopted internationally in order to accommodate emerging variants and, at the same time, constrain the number and depth of hierarchical lineage labels [4]. Analysis of viral genomes—isolated from affected subjects from many countries—has allowed the identification of eight major clades: L (including the first Chinese cases reported), S, O, V, G and its derivatives GH, GV, GR and GRY [5,6,7]. G and its derivatives became the dominant clades (by far) worldwide around March 2020. The G clade is defined by the D614G mutation within the gene encoding the Spike (S) protein that binds its receptor in mammalian cells during infection. The D614G mutant virus displays greater infectivity and is associated with greater viral loads [8]. Furthermore, SARS-CoV-2 D614G also shows enhanced replication ex vivo and earlier transmission by in vivo experiments [9].

As of March 2021, the GR clade (lineage B.1.1.1) and its variants (including B.1.1.7, first observed in the United Kingdom and B.1.1.28, identified in Brazil) are predominant worldwide (GISAID database; http://www.gisaid.org; accessed on 30 March 2021). This clade is defined by an insertion/deletion GGG > AAC within the open reading frame (ORF) encoding the N (nucleocapsid) protein, replacing an arginine and a glycine residue in positions 203 and 204 for a lysine and an arginine residue, respectively. Hence, it is termed the 203_204delinsKR mutation (coordinates nt28881-28881) [5,6].

The genotyping of clades and related variants can be useful for monitoring the population evolution of the pandemic, validating transmission routes, and determining their association with different clinical variables or outcomes [10]. Different next-generation sequencing (NGS) experimental designs have been implemented to study the genomic diversity of SARS-CoV-2 worldwide [11,12], and they can be broadly classified in three main groups: (i) metagenomics (specifically, sequencing aimed at all the RNA or cDNA species present in a sample) and the subsequent identification of viral sequences using bioinformatic approaches [13,14]; (ii) probe-based target enrichment, a strategy that selectively captures SARS-CoV-2-derived library fragments by means of hybridization to a set of probes [15] and; iii) amplicon-based enrichment, where SARS-CoV-2 sequences are selectively amplified by PCR from RNA sample-derived cDNAs, to generate overlapping fragments [16,17]. However, these approaches require highly specialized equipment and personnel that are not readily available in many highly populated territories [18].

Short-amplicon high-resolution melting analysis (SA-HRM) is a post-PCR, closed-tube method that can be performed using more widely available qPCR equipment, as well as dedicated instrumentation. SA-HRM allows cost-effective genotyping of single-nucleotide differences—as well as other types of variants—and relies on the differential melting profile of a short double-stranded DNA molecule in the presence of an intercalating agent [19].

Here, we report, for the first time, the genotyping of a SARS-CoV-2 clade (GR) by short-amplicon high-resolution melting analysis.

## 2. Materials and Methods

### 2.1. Subjects and Samples

After informed consent and study approval by the institutional ethics and biosafety committees, saliva samples were obtained as previously reported by us [20] from fourteen patients showing RT-PCR positive, as well as a sample from a patient negative for SARS-CoV-2, according to a standard TaqMan qPCR assay of three amplicons within the RdRp (RNA-dependent RNA polymerase gene), E (envelope protein gene) and N (nucleocapsid protein gene) [21]. Total RNA was isolated using the QIAamp Viral RNA Mini Kit (Qiagen, Hilden, Germany) according to directions provided by the manufacturer. Spectrophotometer UV readings at 260 and 280 nm were used to estimate concentration (260 nm) and purity (260/280 ratio) of the RNA samples.

### 2.2. cDNA Synthesis

360 ng of purified total RNA isolated from every saliva sample were retrotranscribed using the GoScript Reverse Transcriptase kit (Promega Corporation, Madison, WI, USA) with random hexamers, following manufacturer’s instructions, to generate 20 µL reactions. The cDNAs samples obtained were employed for SA-HRM and ARMS assays as well as Sanger sequencing.

### 2.3. Small-Amplicon High-Resolution Melting Analysis (SA-HRM)

An amplicon of 54 bp (Table 1) was designed for the genotyping of samples belonging to the GR clade of the SARS-CoV-2 virus, using the GR-SA-HRM-F and GR-SA-HRM-R primers. The 3߰ end of the upstream (forward) primer was located 5 bp form the GGG/AAC insertion/deletion whereas the 3߰ of the downstream (reverse) primer was located 1 bp from the insertion/deletion. This asymmetry was needed to ensure a similar optimal annealing temperature for both primers, in order to achieve a robust PCR amplification. 1 µL of cDNA reactions—corresponding to the RNA samples and a negative control—were amplified by PCR with both primers at a final concentration of 200 nM and an initial denaturation step at 95 °C for 3 min, followed by 29 cycles, each with denaturation at 95 °C for 5 s, annealing at 61 °C for 15 s and extension at 70 °C for 10 s. A premelt was performed with a ramp of 20 °C/s, initiating at 95 °C and ending at 37 °C. The melting step (0.3 °C/s) was initiated at 55 °C and ended at 95 °C. The PCR, premelt and melting were carried out using a LightScanner 32 Instrument (Biofire Defense, Salt Lake City, UT), employing the LightScanner Master Mix, containing the intercalating agent LCGreen (Biofire Defense). Traces belonging to the saliva RNA from a SARS-CoV-2 negative control, as well as the negative control, were removed once it was verified that they showed no significant peaks in –(d/dT) relative fluorescence/temperature plots obtained during the SA-HRM. The remaining traces were normalized in the X-axis interval between 78.04 °C and 85.14 °C, establishing a sensibility value of −3.00.

### 2.4. Amplification-Refractory Mutation System (ARMS)

An amplification-refractory mutation system (ARMS) assay [22] was designed (Table 1) to selectively amplify cDNA generated from viruses belonging or not belonging to the GR clade—generating 649 bp or 295 bp, respectively. Each PCR reaction was prepared using GoTaq Green Master Mix (Promega, Madison, WI) and contained 1 µL of cDNA as well as the GR-ARMS-F, GR-ARMS-R, non-GR-ARMS-F and non-GR-ARMS-R primers, each at a final concentration of 300 nM. An initial denaturation step at 94 °C for 3 min was followed by 40 cycles, each consisting of a denaturation step at 94 °C for 30 s, an annealing step 55 °C for 25 s and an extension step 68 °C for 40 s. A final extension was carried out at 68 °C for 3 min. 7 µL of the PCR products were run in 3% agarose gels. The PCR amplification, the agarose gel electrophoresis and the gel documentation were conducted entirely using the miniPCR DNA Discovery System (MiniPCR, Cambridge, MA, USA). Optical densitometry data of the gel bands were obtained using ImageJ software (NIH, Bethesda, MD, USA). Optical density values from individual SARS-CoV-2-derived bands were normalized by expressing them as ratios of the optical density value of the 350 bp band included in the molecular weight marker.

### 2.5. Sanger Sequencing

An amplicon of 304 bp was designed (Table 1) for PCR amplification of a segment of cDNA spanning the 203_204delinsKR mutation using the GR-Sanger-F and GR-Sanger-R primers. 10 µL of the cDNA reaction were PCR-amplified using GoTaq Green Master Mix in 100 µL reactions with both primers at a final concentration of 300 nM, and an initial denaturation step at 94 °C for 3 min, followed by 40 cycles, each consisting of a denaturation step at 94 °C for 30 s, an annealing step at 57 °C for 30 s and an extension step 68 °C for 20 s. A final extension was carried-out at 68 °C for 5 min. The PCR products were purified from excised 2.5% agarose gel bands using the QIAquick Gel Extraction Kit (Qiagen) and quantified with a Nanodrop spectrophotometer (Thermo-Scientific, Waltham, MA, USA). 100 ng of purified PCR product was used to generate 20 µL Sanger sequencing reactions with the BigDye Terminator kit v3.1. All four PCR products were sequenced bidirectionally, employing the amplifying primers (GR-Sanger-F and GR-Sanger-R) for this purpose. Sequencing reactions were, in turn, purified employing Centri-Sep columns (Thermo-Scientific). Capillary electrophoreses were run in a Genetic Analyzer 310 (Thermo-Scientific) following manufacturer’s instructions.

### 2.6. Statistical Analyses

Descriptive statistics consisted in calculation of mean and standard deviation (SD). Parametric analyses were performed using Student’s *t* test for nonpaired samples, with 95% confidence intervals (CI) and normality of residuals evaluated using Shapiro’s test (*p* > 0.05). Correlation was assessed using Pearson’s test. Nonparametric analyses were performed by means of the Mann–Whitney U test. Nonexistent values were not considered for these analyses. All statistical images and calculations were processed using R language version 3.6.2.

## 3. Results

The main features of the fourteen SARS-CoV-2 positive RNA samples are summarized in Table 2.

**Figure 1:**
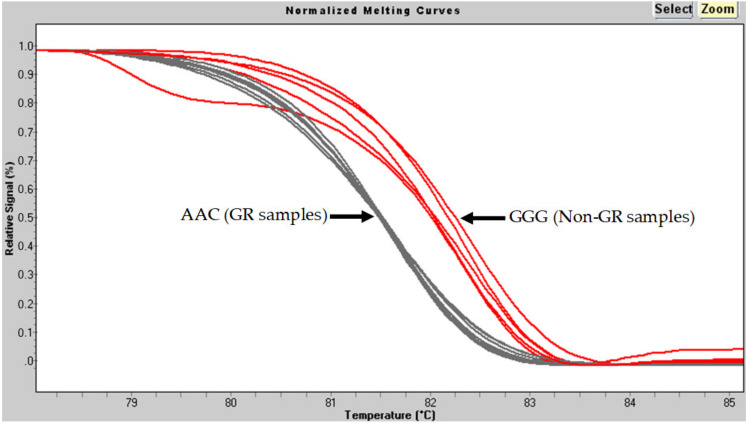
Normalized SA-HRM traces corresponding to the SARS-CoV-2 positive samples within relative fluorescence/temperature plots forming two clearly distinctive groups.

**Figure 2:**
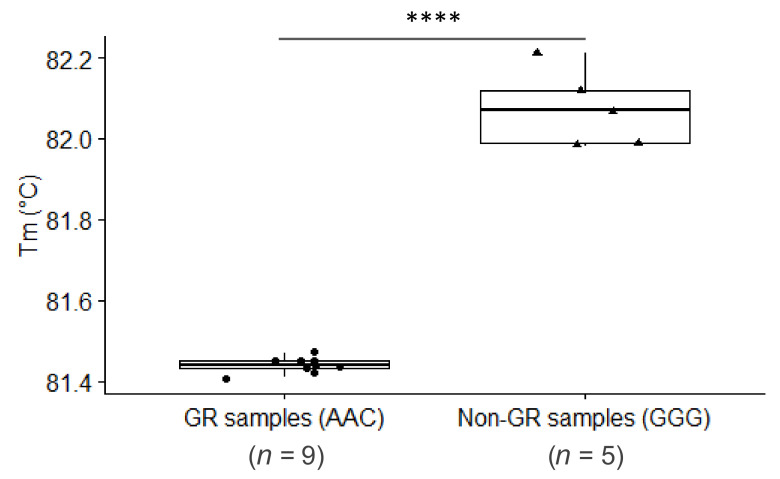
Differences between the melting temperatures (Tm) of samples forming two distinctive groups according to the denaturation profiles, showing a statistically significant difference (**** *p* = 9.67 × 10^−5^, CI: −0.75 to −0.51 °C).

The normalized SA-HRM traces corresponding to the SARS-CoV-2 positive samples within the relative fluorescence/temperature plot (Figure 1) formed two clearly distinctive groups. According to the shape of the traces, the group showing earlier denaturation (9 samples) was predicted to represent SARS-CoV-2 belonging to GR samples (AAC, fewer hydrogen bonds between the two DNA strands), whereas the group with later denaturation (5 samples) was predicted to represent viruses not belonging to GR samples (GGG, more hydrogen bonds). A statistically significant difference was observed between the melting temperatures of both groups (*p* = 9.67 × 10^−5^, CI: −0.75 to −0.51 °C) (Figure 2). The nonnormalized SA-HRM –(d/dT)/temperature traces indicated that non-GR samples generated weaker signals during the denaturation process (see Figure S1).

**Figure 3:**
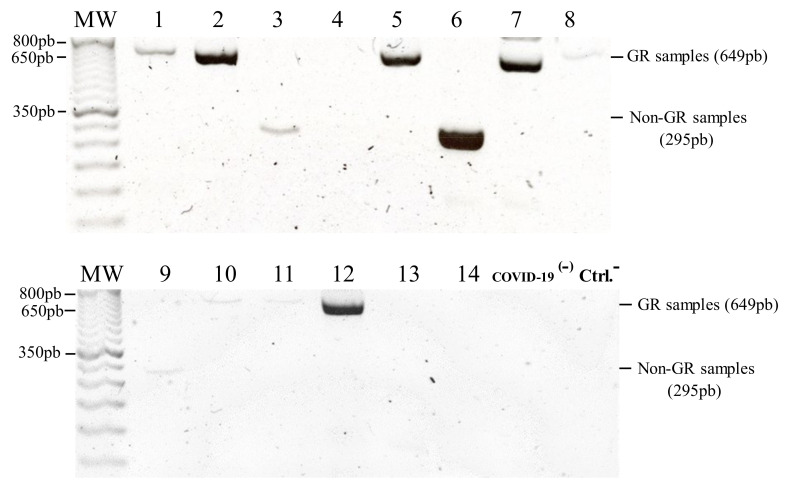
Agarose gels showing the electrophoresis of the ARMS assay products. The bands corresponding to molecules of 649 bp indicate the presence of GR clade samples whereas bands of 295 bp indicate non-GR samples. Note that samples 4, 13 and 14 did not yield a distinguishable product. (MW, molecular weight marker; 1-14, SARS-CoV-2 positive samples; COVID-19^(−)^, SARS-CoV-2 negative saliva RNA sample; Ctrl−, Negative, no-cDNA control).

Each of the ten samples that yielded a distinguishable product in the ARMS assay was classified according to size (649 bp for the GR clade and 295 bp for the non-GR samples), consistent with the grouping suggested by the SA-HRM assay (Figure 3 and Table 3). Optical densitometry of the bands obtained through the ARMS assay showed that band intensity was significantly correlated to RNA sample concentration and purity, but not correlated to Ct of viral TaqMan amplicons (Table 4).

**Figure 4:**
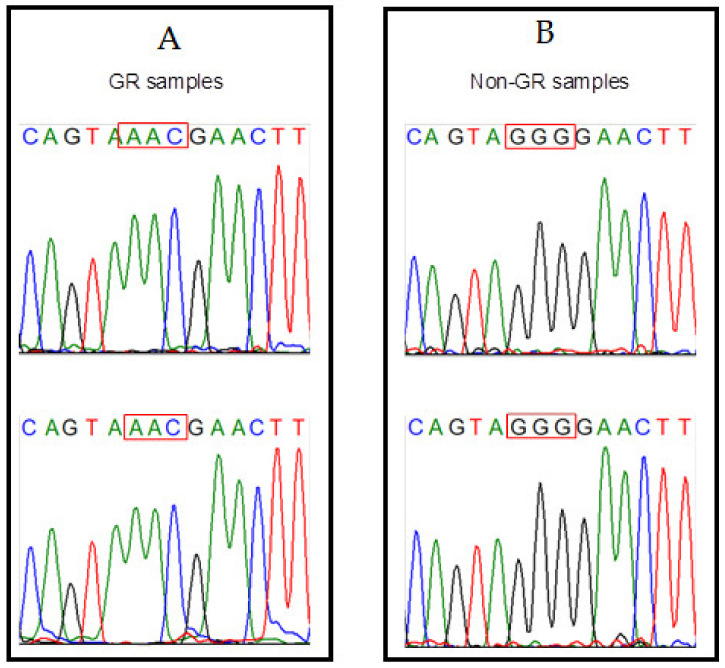
Traces obtained from four Sanger sequencing reactions of randomly selected samples predicted to belong to the GR samples ((**A**), *n* = 2) and non-GR samples ((**B**), *n* = 2).

Sanger sequencing of four randomly selected samples—two classified as GR and two classified as non-GR—was also consistent with the SA-HRM data (Figure 4). Legible adjacent segments at either side of the insertion/deletion did not reveal additional variation in comparison with the canonical sequence.

Interestingly, we observed that samples belonging to the GR clade had significantly smaller Ct values corresponding to the TaqMan amplicons of SARS-CoV-2 (Table 5).

## 4. Discussion 

We present herein the implementation of two molecular biology methods (SA-HRM and ARMS) for the typification of a SARS-CoV-2 mutation that defines the GR clade.

This SA-HRM approach could be adapted to screen for variants of current interest, such as B.1.351 (first observed in South Africa) [23] or the P.1 variant (identified in Japan in travelers from Brazil) [24]. Specifically, within the receptor binding domain (RBD) of the Spike protein, position 417 can be occupied by an asparagine (N) amino acid residue in the B.1.351 variant, by a threonine (T) in the P.1 variant and by a lysine (K) residue in SARS-CoV-2 virus without RBD mutations. A modification of our SA-HRM assay can be thus employed to differentiate between the three situations described above, locating the primers around the 417 codon of the Spike ORF.

In terms of our SA-HRM assay, the differential quantity of hydrogen bonds between complementary strands of the PCR products predicted a melting pattern with earlier denaturation of GR sample-derived molecules and later denaturation of non-GR-derived molecules. That pattern was observed, and the sample classification was validated by means of the ARMS assay and Sanger sequencing.

Interestingly, we observed that, in our relatively reduced group of subjects, GR samples showed significantly lower Ct values in the diagnostic qRT-PCR assays in comparison to non-GR samples (Table 5). This suggests that there was a smaller quantity of starting material in the non-GR SA-HRM PCRs, which could result in less robust and therefore more variable amplification. Additionally, the nonnormalized SA-HRM –(d/dT)/temperature traces showed that non-GR samples generated weaker signals during melting (see Figure S1). These two factors could explain the more variable SA-HRM traces of the non-GR samples upon normalization (Figure 1).

Although it was possible to classify all of our samples using the SA-HRM assay, three of them did not generate a band in the ARMS assay gel that could allow classification (Figure 3 and Table 3). We observed that, in the ARMS gel, the bands showed a broad range of variation and that optical density of the bands was positively correlated with RNA concentration and negatively correlated with the ratio of absorbance at 260 and 280 nm. Traditionally, it has been considered that a 260/280 ratio of 2.0 indicates a RNA sample with high purity [25]. The RNA isolation kit used employed carrier RNA and an elution buffer that contained sodium azide. It has been shown that both the carrier RNA and the sodium azide can increase the 260/280 ratio significantly [26]. Additionally, the length of the SA-HRM was 54 bp, whereas the ARMS amplicons were 295 bp and 649 bp long. RNA integrity, which allows synthesis of longer cDNAs could, therefore, be a factor that renders the SA-HRM assay suitable for a broader range of sample quality specifications in comparison to the ARMS assay.

## 5. Conclusions

Due to the proofreading enzymatic activity encoded by the SARS-CoV-2 genome, single nucleotide substitutions occur at a slower rate in comparison to other RNA viruses [27]. However, deletions are not subject to this control mechanism [28]. It has also been shown that recurrent deletions within the viral genomic segment encoding the Spike protein confer resistance to neutralizing antibodies [28], therefore potentially diminishing the effectiveness of vaccines. Given its nature, our SA-HRM assay is particularly suited to genotype deletions, a feature that can be used to screen for strains evading active or passive immunity.

Like the PCR amplification, the agarose gel electrophoreses and gel documentation corresponding to our ARMS assay were conducted using the miniPCR DNA Discovery System (see Materials and Methods), an instrument that currently costs under 800 USD. We consider that this represents an alternative for SARS-CoV-2 variant typing of specific mutations in territories with limited equipment availability.

## Figures and Tables

**Table 1:** Specifications of primers and PCR assays employed for the short-amplicon high-resolution melting analysis (SA-HRM), amplification-refractory mutation system (ARMS) and Sanger experiments. Bases in bold highlight the clade discrimination segments of the ARMS primers.

Assay	Primer	Sequence (5′ → 3′) Position at SARS-CoV-2 Isolate Wuhan-Hu-1 * Position at SARS-CoV-2 N gene: NC_045512.2 **	Annealing Temperature (°C)	Cycles	Product Size (bp)
SA-HRM	GR-SA-HRM-F	CAAGAAATTCAACTCCAGGCAG (nt28854 → 28875) * (nt581 → 602) **	61	29	54
	GR-SA-HRM-R	CAGCCATTCTAGCAGGAGAAGTT (nt28907 → 28885) * (nt634 → 612) **			
ARMS	GR-ARMS-F	ACTCCAGGCAGCAGTA**AAC** (nt28865 → nt28883) * (nt592 → 610) **	55	40	649
	GR-ARMS-R	CACTGCTCATGGATTGTTG (nt29513 → 29495) * (nt1240 → 1222) **			
	non-GR-ARMS-F	CTACCTAGGAACTGGGCCAG (nt28606 → 28625) * (nt333 → 352) **	55	40	295
	non-GR-ARMS-R	TCTAGCAGGAGAAGTTC**CCC** (nt28900 → 28881) * (nt627 → 608) **			
Sanger	GR-Sanger-F	CAAAGACGGCATCATATGG (nt28651 → 28669) * (nt378 → 396) **	57	40	304
	GR-Sanger-R	CAATCTGTCAAGCAGCAGC (nt28954 → 28936) * (nt681 → 663) **			

* Denotes coordinates within SARS-CoV-2 Isolate Wuhan-Hu-1; ** Denotes coordinates within SARS-CoV-2 N gene (Accession number: NC_045512.2).

**Table 2:** Main features of the SARS-CoV-2 positive RNA samples employed in the cDNA synthesis.

Feature	Mean ± SD (*n* = 14)
Concentration (ng/μL)	105.64 ± 23.34
Purity (260/280)	3.21 ± 0.31
qPCR Ct E	20.74 ± 6.92
qPCR Ct RdRp	20.07 ± 4.39
qPCR Ct N	21.10 ± 5.87

**Table 3:** Clade GR or non-GR classification of the SARS-CoV-2 positive samples (SPL), according to the SA-HRM and ARMS assays.

Sample	Assay	
	SA-HRM	ARMS
SPL-1	GR	GR
SPL-2	GR	GR
SPL-3	Non-GR	Non-GR
SPL-4	Non-GR	-
SPL-5	GR	GR
SPL-6	Non-GR	Non-GR
SPL-7	GR	GR
SPL-8	GR	GR
SPL-9	Non-GR	Non-GR
SPL-10	GR	GR
SPL-11	GR	GR
SPL-12	GR	GR
SPL-13	GR	-
SPL-14	Non-GR	-

**Table 4:** Correlation of normalized optical density of bands resulting from the ARMS assay to concentration and purity of the RNA samples, as well as Ct values corresponding to real-time RT-PCR assays of the RdRp, E and N SARS-CoV-2 genes.

	r (Relative Amplification) (*n* = 14)	Confidence Intervals (95%)		*p*-Value
		Lower	Upper	
Concentration (ng/μL)	0.84	**0.3777099**	**0.9450479**	**0.00331**
Purity (260/280)	−0.828	**−0.9449925**	**−0.3772663**	**0.003323**
PCR Ct E	−0.586	−0.7877534	0.3098872	0.2823
PCR Ct RdRp	−0.351	−0.8033255	0.3572016	0.3193
PCR Ct N	−0.504	−0.7711625	0.3476024	0.3394

Statistically significant values are shown in bold.

**Table 5:** Comparison of parameters between the GR samples and non-GR samples according to the SA-HRM assay.

	GR Samples	Non-GR Samples	*p*-Value
	Mean ± SD (*n* = 9)	Mean ± SD (*n* = 5)	
Concentration (ng/μL)	106.38 ± 25.67	104.29 ± 21.19	1
Purity (260/280)	3.16 ± 0.31	3.28 ± 0.34	0.5185
PCR Ct E	17.15 ± 2.25	27.22 ± 7.99	**0.006993**
PCR Ct RdRp	18.12 ± 2.03	24.46 ± 5.38	**0.01119**
PCR Ct N	18.05 ± 1.78	26.59 ± 6.87	**0.006993**
Relative amplification	1.60 ± 1.71	2.29 ± 3.19	0.9273

Statistically significant values are shown in bold.

## Data Availability

All data generated by this work is freely available upon request.

## Supplementary Materials

The following are available online at https://www.mdpi.com/article/10.3390/genes12040531/s1, Figure S1: Non-normalized –(d/dT)/temperature SA-HRM traces.

## References

[1] Hu B., Guo H., Zhou P., Shi Z.-L. (2020). Characteristics of SARS-CoV-2 and COVID-19. Nat. Rev. Microbiol..

[2] Lauring A.S., Hodcroft E.B. (2021). Genetic Variants of SARS-CoV-2—What Do They Mean?. JAMA.

[3] Grubaugh N.D., Petrone M.E., Holmes E.C. (2020). We shouldn’t worry when a virus mutates during disease outbreaks. Nat. Microbiol..

[4] Rambaut A., Holmes E.C., O’Toole Á., Hill V., McCrone J.T., Ruis C., du Plessis L., Pybus O.G. (2020). A dynamic nomenclature proposal for SARS-CoV-2 lineages to assist genomic epidemiology. Nat. Microbiol..

[5] Mercatelli D., Giorgi F.M. (2020). Geographic and Genomic Distribution of SARS-CoV-2 Mutations. Front. Microbiol..

[6] Koyama T., Platt D., Parida L. (2020). Variant analysis of SARS-CoV-2 genomes. Bull. World Health Organ..

[7] Arena F., Pollini S., Rossolini G.M., Margaglione M. (2021). Summary of the Available Molecular Methods for Detection of SARS-CoV-2 during the Ongoing Pandemic. Int. J. Mol. Sci..

[8] Korber B., Fischer W.M., Gnanakaran S., Yoon H., Theiler J., Abfalterer W., Hengartner N., Giorgi E.E., Bhattacharya T., Foley B. (2020). Tracking Changes in SARS-CoV-2 Spike: Evidence that D614G Increases Infectivity of the COVID-19 Virus. Cell.

[9] Hou Y.J., Chiba S., Halfmann P., Ehre C., Kuroda M., Dinnon K.H., Leist S.R., Schäfer A., Nakajima N., Takahashi K. (2020). SARS-CoV-2 D614G variant exhibits efficient replication ex vivo and transmission in vivo. Science.

[10] Martin M.A., VanInsberghe D., Koelle K. (2021). Insights from SARS-CoV-2 sequences. Science.

[11] Xiao M., Liu X., Ji J., Li M., Li J., Yang L., Sun W., Ren P., Yang G., Zhao J. (2020). Multiple approaches for massively parallel sequencing of SARS-CoV-2 genomes directly from clinical samples. Genome Med..

[12] WHO (2021). Genomic Sequencing of SARS-CoV-2: A Guide to Implementation for Maximum Impact on Public Health, 8 January 2021.

[13] Van Tan L., Man D.N.H., Hang V.T.T., Khanh P.N.Q., Xuan T.C., Phong N.T., Tu T.N.H., Hien T.T., Hung L.M., Truong N.T. (2020). SARS-CoV-2 and co-infections detection in nasopharyngeal throat swabs of COVID-19 patients by metagenomics. J. Infect..

[14] Bal A., Destras G., Gaymard A., Bouscambert-Duchamp M., Valette M., Escuret V., Frobert E., Billaud G., Trouillet-Assant S., Cheynet V. (2020). Molecular characterization of SARS-CoV-2 in the first COVID-19 cluster in France reveals an amino acid deletion in nsp2 (Asp268del). Clin. Microbiol. Infect..

[15] Wen S., Sun C., Zheng H., Wang L., Zhang H., Zou L., Liu Z., Du P., Xu X., Liang L. (2020). High-coverage SARS-CoV-2 genome sequences acquired by target capture sequencing. J. Med. Virol..

[16] Bhoyar R.C., Jain A., Sehgal P., Divakar M.K., Sharma D., Imran M., Jolly B., Ranjan G., Rophina M., Sharma S. (2021). High throughput detection and genetic epidemiology of SARS-CoV-2 using COVIDSeq next-generation sequencing. PLoS ONE.

[17] Harilal D., Ramaswamy S., Loney T., Suwaidi H.A., Khansaheb H., Alkhaja A., Varghese R., Deesi Z., Nowotny N., Alsheikh-Ali A. (2020). SARS-CoV-2 Whole Genome Amplification and Sequencing for Effective Population-Based Surveillance and Control of Viral Transmission. Clin. Chem..

[18] Helmy M., Awad M., Mosa K.A. (2016). Limited resources of genome sequencing in developing countries: Challenges and solutions. Appl. Transl. Genom..

[19] Liew M., Pryor R., Palais R., Meadows C., Erali M., Lyon E., Wittwer C. (2004). Genotyping of Single-Nucleotide Polymorphisms by High-Resolution Melting of Small Amplicons. Clin. Chem..

[20] López-Martínez B., Guzmán-Ortiz A.L., Nevárez-Ramírez A.J., Parra-Ortega I., Olivar-López V.B., Ángeles-Floriano T., Vilchis-Ordoñez A., Quezada H. (2020). Saliva as a promising biofluid for SARS-CoV-2 detection during the early stages of infection. Bol. Med. Hosp. Infant. Mex..

[21] Corman V.M., Landt O., Kaiser M., Molenkamp R., Meijer A., Chu D.K., Bleicker T., Brünink S., Schneider J., Schmidt M.L. (2020). Detection of 2019 novel coronavirus (2019-nCoV) by real-time RT-PCR. Euro Surveill..

[22] Little S. (1995). Amplification-Refractory Mutation System (ARMS) Analysis of Point Mutations. Curr. Protoc. Hum. Genet..

[23] Walensky R.P., Walke H.T., Fauci A.S. (2021). SARS-CoV-2 Variants of Concern in the United States—Challenges and Opportunities. JAMA.

[24] Toovey O.T.R., Harvey K.N., Bird P.W., Tang J.W.-T.W.-T. (2021). Introduction of Brazilian SARS-CoV-2 484K.V2 related variants into the UK. J. Infect..

[25] Wilfinger W.W., Mackey K., Chomczynski P. (1997). Effect of pH and ionic strength on the spectrophotometric assessment of nucleic acid purity. Biotechniques.

[26] El Bali L., Diman A., Bernard A., Roosens N.H.C., De Keersmaecker S.C.J. (2014). Comparative study of seven commercial kits for human DNA extraction from urine samples suitable for DNA biomarker-based public health studies. J. Biomol. Tech. JBT.

[27] Minskaia E., Hertzig T., Gorbalenya A.E., Campanacci V., Cambillau C., Canard B., Ziebuhr J. (2006). Discovery of an RNA virus 3’→5’ exoribonuclease that is critically involved in coronavirus RNA synthesis. Proc. Natl. Acad. Sci. USA.

[28] McCarthy K.R., Rennick L.J., Nambulli S., Robinson-McCarthy L.R., Bain W.G., Haidar G., Duprex W.P. (2021). Recurrent deletions in the SARS-CoV-2 spike glycoprotein drive antibody escape. Science.

