# Supplementary material for: Genotyping of the Major SARS-CoV-2 Clade by Short-Amplicon High-Resolution Melting (SA-HRM) Analysis
Source: Genes (Basel). 2021 Apr 5;12(4):531. doi: 10.3390/genes12040531 (PMC8067340; doi:10.3390/genes12040531)

Supplementary Figure 1: Non-normalized –(d/dT)/temperature SA-HRM traces, full length [A]. The peaks corresponding to non-GR samples (in red, shifted to the right in the images below and identified as such after normalization, amplified to better visualization in [B]) are smaller than the peaks of the GR samples (in gray, shifted to the left). This means that the signal generated upon de denaturation of the non-GR PCR products generates a weaker signal in comparison to the GR PCR products, hence the increased noise of that group upon normalization.

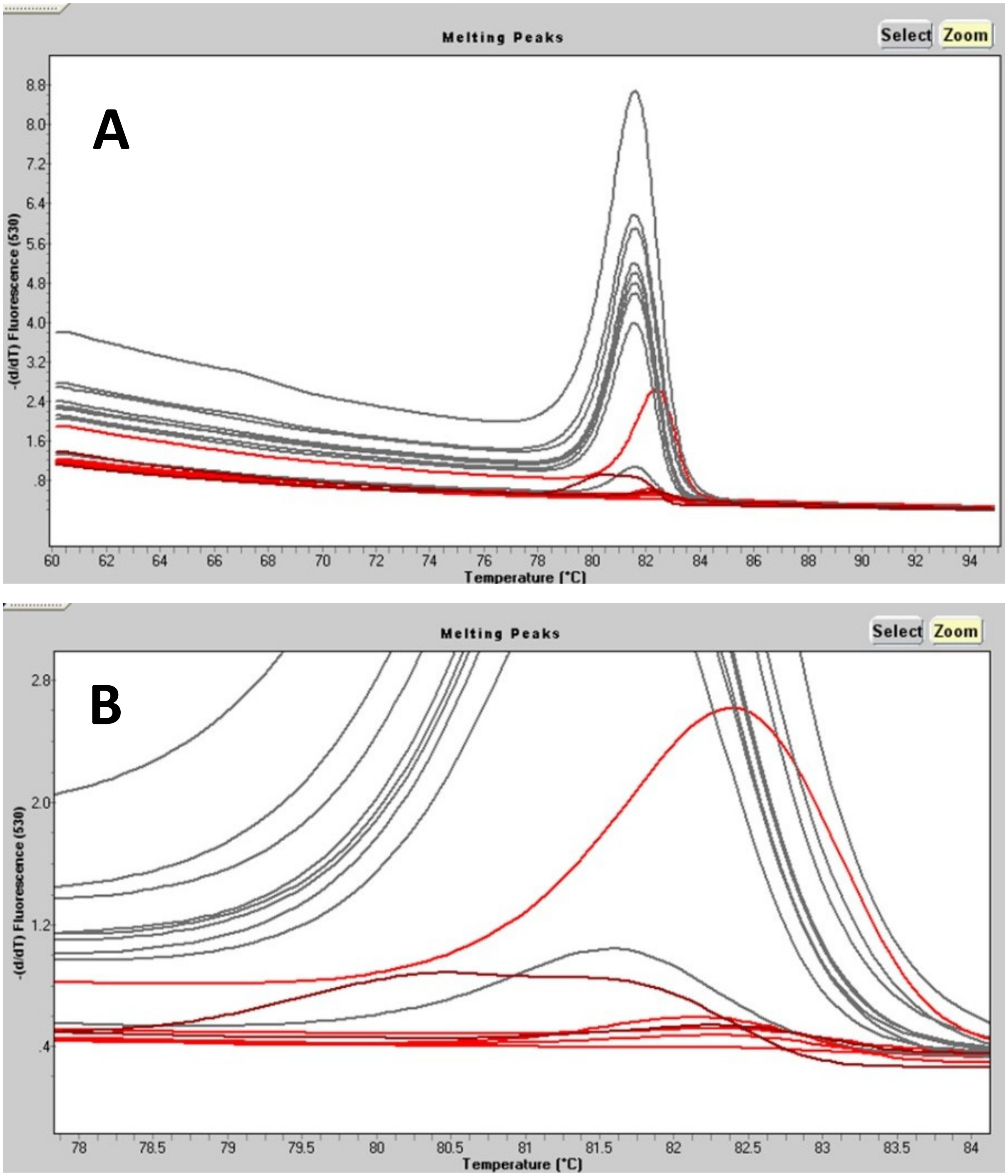

Supplement: Supplementary file 1 [file genes-12-00531-s001.pdf]
